# Oral and hand hygiene practices among adolescents aged 12–17 years in 72 countries: a cross-sectional study using global school-based student health survey data (2010–2019)

**DOI:** 10.3389/fpubh.2026.1939346

**Published:** 2026-09-10

**Authors:** Zongwei Huang, Jingru Lin

**Affiliations:** Beijing University of Chinese Medicine Shenzhen Hospital (Longgang), Shenzhen, China

**Keywords:** adolescents, Global School-based Student Health Survey, hand hygiene, health disparities, toothbrushing

## Abstract

**Background:**

Oral and hand hygiene constitute fundamental determinants of adolescent health. However, globally comparable estimates of these behaviours remain limited. We aimed to estimate the prevalence of toothbrushing and handwashing practices among adolescents aged 12–17 years and to quantify disparities by sex, age, geographical region, and national income level.

**Methods:**

We used nationally representative data from the Global School-based Student Health Survey (GSHS) collected between 2010 and 2019, covering 244,040 adolescents from 72 countries. Toothbrushing frequency was categorised as <1 time/day, 1 time/day, and ≥2 times/day. Hand hygiene was assessed via three items: never or rarely washing hands before eating, after using the toilet, and using soap. The overall, subgroup-specific, and country-specific prevalence estimates were calculated accounting for primary sampling units, strata, and sampling weights.

**Results:**

The overall prevalence of brushing teeth <1 time/day was 10.9% (95%CI = 10.0–11.8), ranging from 2.6% in Benin to 35.2% in Yemen. Brushing ≥2 times/day was reported by 70.5% (68.8–72.0). Never or rarely washing hands before eating, after toilet use, and using soap were reported by 6.5% (6.1–7.0), 5.5% (5.1–5.9), and 9.0% (8.5–9.5), respectively. Boys exhibited inferior hygiene practices relative to girls (for instance, brushing <1 time/day: 13.1% vs. 8.6%; never/rarely using soap: 10.4% vs. 7.6%). Younger adolescents (12–14 years) showed a higher prevalence of inadequate brushing (12.2% vs. 9.4%) but marginally better handwashing before eating than older adolescents (15–17 years). The Eastern Mediterranean Region displayed the highest prevalence of inadequate brushing (30.7%), whereas the Region of the Americas showed the lowest (5.3%). A marked economic gradient was observed for toothbrushing (inadequate brushing declined from 12.5% in the poorest quintile to 4.4% in the richest), but hand hygiene showed no monotonic income gradient.

**Conclusion:**

Inadequate oral and hand hygiene practices remain prevalent among adolescents globally, with substantial heterogeneity by sex, age, region, and national income. Targeted school-based interventions, particularly for boys and in low-income and Eastern Mediterranean countries, are urgently needed.

## Introduction

Oral diseases affect an estimated 3.5 billion people worldwide, with untreated dental caries representing the most prevalent condition (1, 2). The World Health Organization (WHO) Global Oral Health Status Report estimated that 2.5 billion people have untreated caries in permanent teeth, with the highest numbers in South-East Asia and the Western Pacific regions (3). Inadequate hand hygiene also contributes substantially to diarrhoeal diseases and respiratory infections, which remain leading causes of morbidity and mortality among children and adolescents in resource-limited settings (4, 5). A systematic review has estimated that washing hands with soap can reduce the risk of diarrhoeal diseases by 42–47% (6).

Adolescence (ages 10–19 years) represents a critical developmental period during which health behaviours are established and consolidated into patterns that persist throughout adulthood (7). Approximately 90% of the world’s adolescents reside in low- and middle-income countries (LMICs), yet the majority of hygiene research has been conducted in high-income settings. Two previous studies have examined toothbrushing using Global School-based Student Health Survey (GSHS) data: McKittrick and Jacobsen analysed 44 LMICs with surveys conducted between 2003 and 2010 (8), and Das Gupta et al. (9) updated this to 72 countries for the period 2010–2019. Both investigations, however, focused exclusively on toothbrushing frequency and did not examine hand hygiene.

For hand hygiene, Jatrana et al. (10) used GSHS data from 92 countries to estimate the prevalence of “appropriate hand hygiene,” defined as washing hands with soap, after using the toilet, and before eating. They reported that only 30.3% of adolescents met this composite standard. However, separate estimates for each handwashing behaviour are not provided, and the composite definition does not identify which specific practice is most deficient. Furthermore, no study has compared the economic gradients of oral and hand hygiene using the same dataset and harmonised analytical methods.

Although oral and hand hygiene constitute low-threshold self-care practices readily incorporated into school-based health promotion, they have historically been targeted through siloed, disease-specific initiatives. Examining both within a single analytical framework allows shared socioeconomic determinants to be elucidated and facilitates the development of integrated hygiene interventions, thereby circumventing the inefficiencies inherent to fragmented, single-behaviour programmes in resource-limited settings. Moreover, purchasing power parity per capita reflects the real economic capacity to afford basic hygiene commodities. Where PPP is low, essential items such as soap, toothpaste, and toothbrushes may represent a substantial proportion of household expenditure, thereby constraining consistent hygiene practice despite their low absolute cost in high-income markets (11).

We therefore used the most recent GSHS data (2010–2019) from 72 countries to: (1) estimate the prevalence of toothbrushing frequency (three categories) and three handwashing practices (never/rarely washing before eating, after toilet use, and using soap); (2) examine disparities by sex, age group (12–14 vs. 15–17 years), WHO region, and country purchasing power parity (PPP) per capita; and (3) identify priority populations for hygiene promotion interventions.

## Methods

### Data source

We used the most recent publicly available GSHS data for each country, collected between 2010 and 2019. The GSHS is a school-based survey developed by WHO and the US Centers for Disease Control and Prevention (CDC) to provide standardised data on health behaviours among adolescents aged 12–17 years (12). The survey employs a two-stage cluster sampling design (schools then classes) to produce nationally representative estimates. All students in selected classes were eligible and completed an anonymous self-administered questionnaire during one class period (40–45 min). The questionnaire was translated into local languages but retained the same core questions across countries. Rigorous back-translation and cognitive testing were conducted to ensure the questionnaire was accurate and error-free across all linguistic versions. Ethical approval was obtained in each country from the Ministry of Health or Education and/or an institutional ethics review board. Written or verbal informed consent was obtained from students and their parents or guardians. The present analysis used publicly available de-identified data and was therefore exempt from additional ethical review. We included all countries that had conducted a GSHS between 2010 and 2019, had available data on toothbrushing and handwashing questions, and had a sample size of at least 100 adolescents aged 12–17 years. A total of 72 countries met the inclusion criteria, representing all six WHO regions except Europe (no European country had toothbrushing data in the most recent survey period), yielding a final sample of 244,040 adolescents. It should be noted that missing data were handled using listwise deletion (complete-case analysis). Among the total survey respondents, 12.7% had missing data on toothbrushing frequency and approximately 12.4% had missing data on each hand hygiene indicator, primarily due to item non-response on the anonymous self-administered questionnaire.

### Outcomes

#### Oral hygiene

Toothbrushing frequency was assessed by the question: “During the past 30 days, how many times per day did you usually clean or brush your teeth?.” Response options were: (1) I did not clean or brush my teeth; (2) Less than 1 time per day; (3) 1 time per day; (4) 2 times per day; (5) 3 times per day; (6) 4 or more times per day. We created three categories: <1 time/day (combining responses 1 and 2), 1 time/day (response 3), and ≥2 times/day (combining responses 4, 5, and 6). This classification follows previous GSHS reports (8, 9).

Hand hygiene was assessed through three specific questions examining: (1) handwashing before eating: “During the past 30 days, how often did you wash your hands before eating?”; (2) handwashing after toilet use (“During the past 30 days, how often did you wash your hands after using the toilet or latrine?”); and (3) soap use (“During the past 30 days, how often did you use soap when washing your hands?”). All items used identical five-point response options: never, rarely, sometimes, most of the time, always. For each question, we defined inadequate hand hygiene as answering “never” or “rarely.” This binary classification follows standard practice in GSHS-based hygiene studies (10).

### Covariates

Covariates included sex, age (dichotomised as 12–14 and 15–17 years), WHO region (Africa, Americas, Eastern Mediterranean, South-East Asia, and Western Pacific), and country economic level. Country economic level was measured using purchasing power parity (PPP) per capita (current international dollars) obtained from the World Bank for the survey year (13). We used PPP per capita rather than World Bank income classifications because PPP more directly reflects household purchasing power and the affordability of basic hygiene commodities. We divided countries into quintiles based on PPP per capita: Q1 (100–3,299 PPP), Q2 (3300–5,999$), Q3 (6000–9,999$), Q4 (10000–17,999$), and Q5 (≥18,000$).

### Statistical analysis

Due to the GSHS two-stage cluster sampling design (schools then classes), we incorporated primary sampling units, strata, and country-specific sampling weights to derive design-based national prevalence estimates and 95% confidence intervals (CIs) for each hygiene indicator. To obtain overall and pooled subgroup estimates (by sex, age group, WHO region, and country income level), we recalculated sampling weights proportional to each country’s sample size (first dividing each original weight by the sum of all original weights, then multiplying the resultant proportion by the sub-population size in each country) (14), thereby ensuring equal contribution across countries and preventing over-representation of populous nations. Prevalence estimates were compared across subgroups using Pearson’s chi-square tests. We additionally fitted multivariable regression models to examine the associations of sex, age group, WHO region, and country-level PPP quintile with each hygiene outcome. Statistical significance was set at *p* < 0.05 (two-sided). All analyses were conducted using SPSS 16.0.

## Results

### Characteristics of the study population

A total of 244,040 adolescents (boys: 50.6%) were included in the analysis. The mean age varied from 13.5 years (Bahamas) to 15.8 years (Laos). Country-specific sample sizes ranged from 134 (Niue) to 25,205 (Malaysia). The overall weighted proportion of adolescents aged 12–14 years was 49.2% and 15–17 years was 50.8% (Table 1).

**Table 1:** Characteristics of the Global School-based Student Health Surveys of young adolescents aged 12–17 years in 72 countries (using the most recent data in each country, 2010–2019).

Country	Survey years	Sample size	Boys (%)	Mean age (years)
Africa				
Algeria	2011	4,342	48.4	14.1
Benin	2016	1,573	68.8	15.5
Eswatini	2013	2,664	44.9	15.3
Ghana	2012	2,366	50.3	14.9
Liberia	2017	1,228	51.4	15.5
Mauritania	2010	1974	54.2	14.8
Mauritius	2019	2,427	46.0	14.8
Mozambique	2015	1,295	51.9	15.0
Namibia	2013	3,313	44.7	15.1
Sierra Leone	2017	2,392	50.4	15.0
Tanzania	2014	3,401	48.9	14.1
Americas				
Bahamas	2013	1,340	48.1	13.5
Barbados	2011	1,617	49.6	14.3
Belize	2011	1968	48.4	14.0
Bolivia	2018	6,823	50.0	15.2
Curacao	2015	2,195	49.8	14.9
Dominican Republic	2016	1,334	50.5	15.2
El Salvador	2013	1857	51.5	14.3
Guatemala	2015	4,208	52.3	14.3
Guyana	2010	2,346	48.7	14.4
Honduras	2012	1715	47.0	13.9
Panama	2018	2,620	47.1	15.1
Paraguay	2017	2,927	48.7	14.8
Peru	2010	2,841	50.4	14.4
Saint Kitts and Nevis	2011	1714	51.3	14.5
Saint Lucia	2018	1852	48.2	14.3
Saint Vincent and the Grenadines	2018	1732	48.0	15.2
Suriname	2016	1893	49.1	14.4
Trinidad and Tobago	2017	3,601	48.2	14.3
Uruguay	2012	3,455	45.9	14.4
Eastern Mediterranean				
Afghanistan	2014	2,165	53.5	14.9
Bahrain	2016	6,856	50.9	14.3
Egypt	2011	2,400	49.8	13.6
Iraq	2012	1980	57.1	14.4
Kuwait	2015	3,297	48.9	15.0
Lebanon	2017	5,172	46.7	14.5
Morocco	2016	5,753	53.0	14.6
Oman	2015	3,130	46.5	15.3
Palestine	2010	14,204	49.9	13.9
Qatar	2011	1822	48.0	13.5
Sudan	2012	2,112	52.9	14.8
Syria	2010	3,034	51.5	13.7
United Arab Emirates	2016	5,404	48.4	14.9
Yemen	2014	2,196	53.7	14.7
South-East Asia				
Bangladesh	2014	2,970	65.3	14.1
Bhutan	2016	6,029	46.1	15.1
Indonesia	2015	10,686	48.9	14.0
Myanmar	2016	2,761	46.3	14.0
Nepal	2015	6,166	48.8	14.3
Sri Lanka	2016	3,213	48.7	14.7
Thailand	2015	5,612	46.8	14.5
Timor-Leste	2015	2,902	48.2	15.4
Western Pacific				
Brunei Darussalam	2019	2,298	50.5	14.6
Cambodia	2013	2,915	50.2	14.9
Cook Islands	2015	649	48.5	15.2
Fiji	2016	3,019	49.4	15.4
French Polynesia	2015	2,864	50.1	14.5
Kiribati	2011	1,555	47.1	14.3
Laos	2015	3,610	53.1	15.8
Malaysia	2012	25,205	50.0	14.9
Mongolia	2013	5,113	48.3	14.5
Nauru	2011	535	46.8	14.2
Niue	2010	134	58.7	14.4
Philippines	2015	8,317	48.6	14.4
Samoa	2017	1,688	47.8	14.8
Solomon Islands	2011	1,293	54.5	14.6
Tokelau	2014	108	56.2	14.1
Tonga	2017	2,858	50.4	14.3
Tuvalu	2013	898	48.5	14.1
Vanuatu	2016	2026	49.4	15.1
Vietnam	2013	3,051	47.0	15.5
Wallis and Futuna	2015	1,027	49.0	14.7
Total		244,040	50.6	14.4

### Toothbrushing frequency

**Figure 1:**
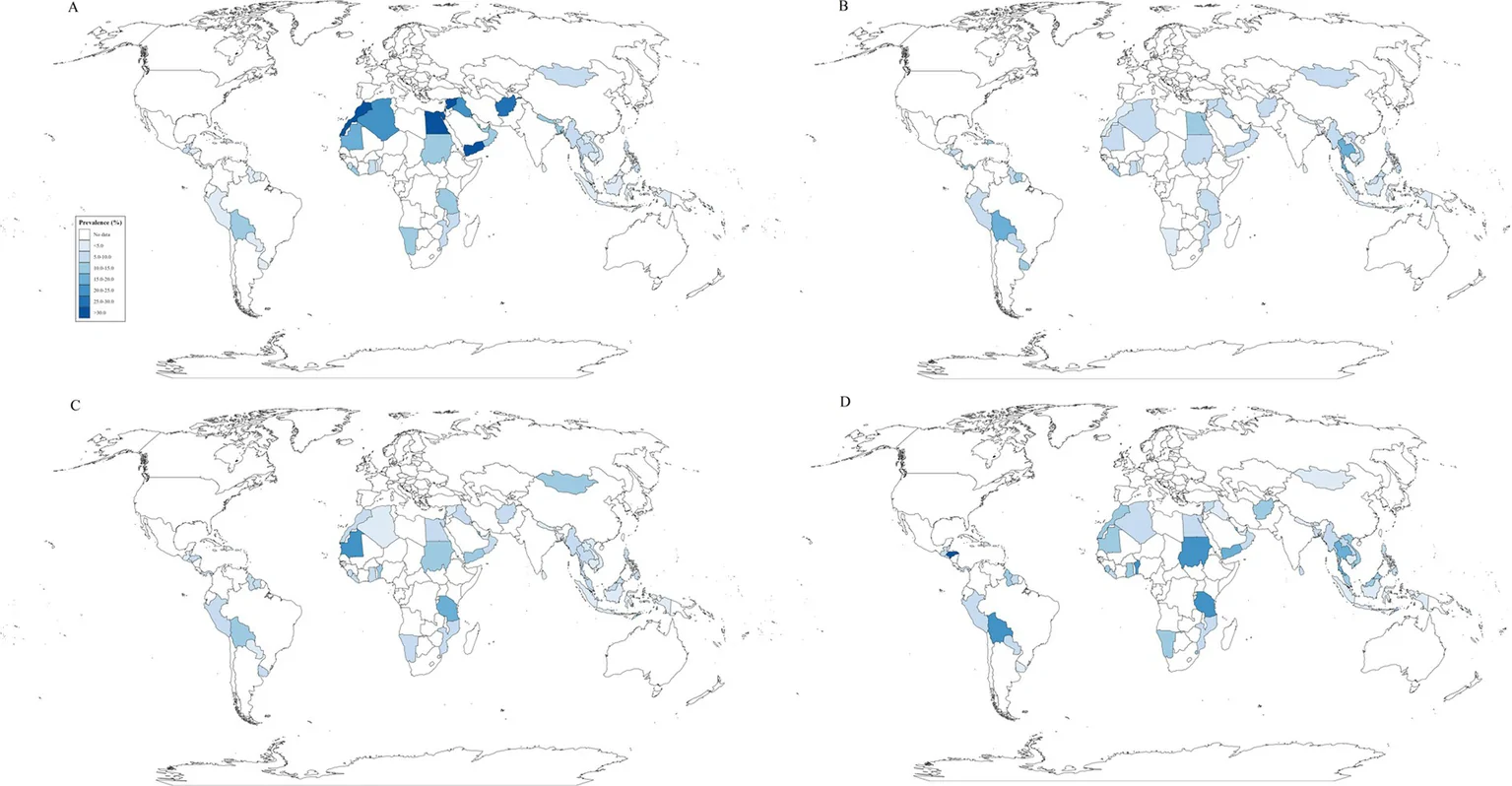
Prevalence of poor oral and hand hygiene in 72 countries. **(A)** Brushing <1 time/day; **(B)** never/rarely washing hands before eating; **(C)** ever/rarely washing hands after toilet use; **(D)** ever/rarely using soap.

**Figure 2:**
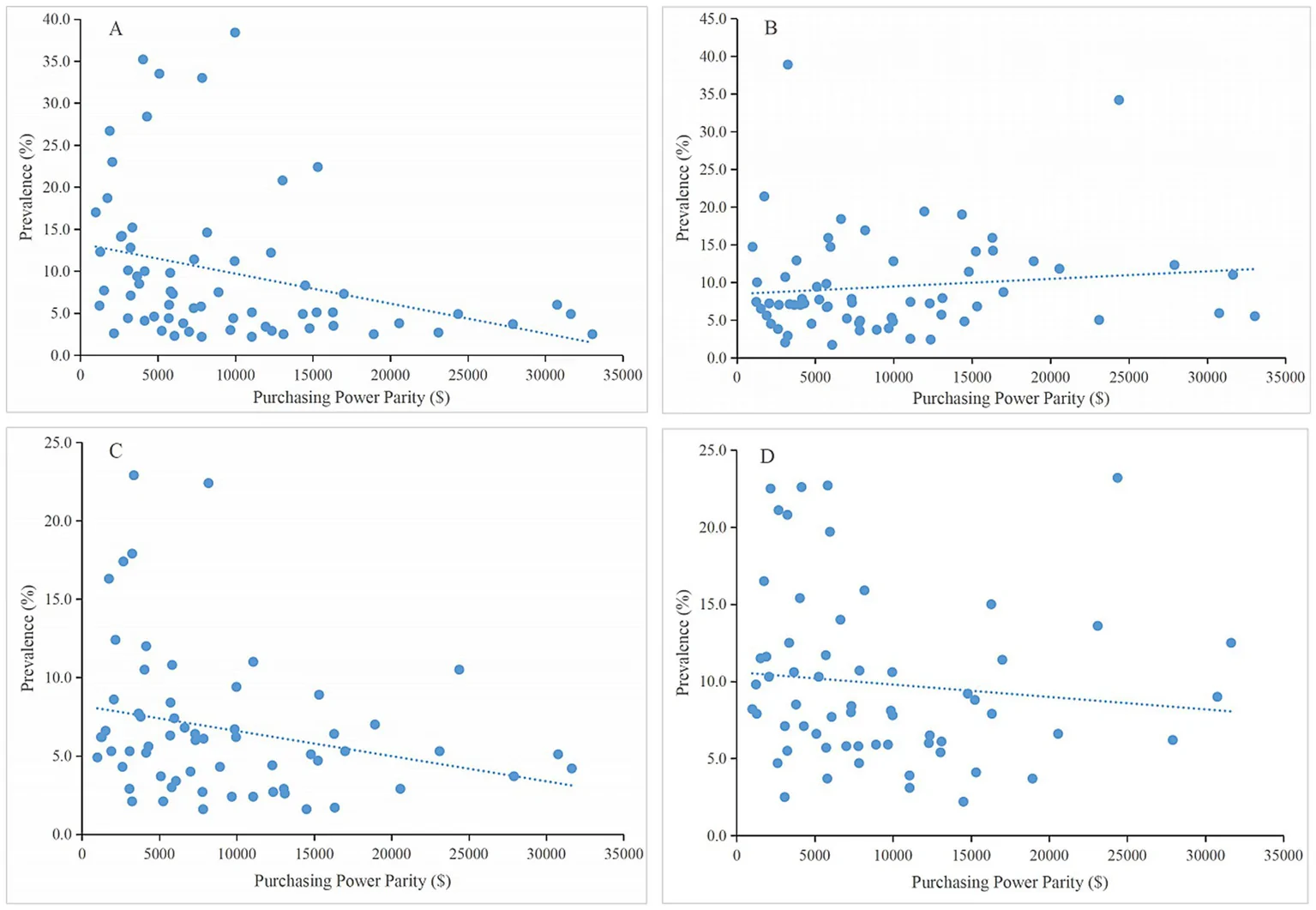
Prevalence of poor oral hygiene by purchasing power parity per capita in 72 countries. **(A)** Brushing <1 time/day; **(B)** never/rarely washing hands before eating; **(C)** never/rarely washing hands after toilet use; **(D)** never/rarely using soap.

Table 2 presents the prevalence of toothbrushing frequency (<1, 1, and ≥2 times/day) among adolescents globally and by sex, age, WHO region, and PPP quintile. Overall, 10.9% (95% CI 10.0–11.8) brushed less than once per day, 18.7% (17.8–19.6) once per day, and 70.5% (68.8–72.0) twice or more per day. Figure 1A shows the between-country heterogeneity in the prevalence of brushing less than once per day. Country-specific estimates for all three frequency categories are provided in Supplementary Table S1, ranging from 2.6% (Benin) to 35.2% (Yemen) for <1 time/day, 5.4% (Guatemala) to 79.7% (Oman) for 1 time/day, and 27.7% (Nauru) to 90.7% (Paraguay) for ≥2 times/day. Boys had a higher prevalence of brushing <1 time/day than girls (13.1% vs. 8.6%), whereas the prevalence of brushing ≥2 times/day was lower in boys (65.7% vs. 75.3%) (Table 2). This pattern was consistent across all WHO regions and PPP quintiles. Younger adolescents (12–14 years) had a higher prevalence of brushing <1 time/day than older adolescents (15–17 years) (12.2% vs. 9.4%), with no significant difference in brushing ≥2 times/day (Table 2). The prevalence varied widely across regions. The Eastern Mediterranean Region had the highest prevalence of brushing <1 time/day (30.7%) and the lowest of brushing ≥2 times/day (42.1%), whereas the Region of the Americas showed the opposite pattern (5.3 and 83.3%, respectively). South-East Asia and the Western Pacific Region showed lower prevalence of brushing <1 time/day and higher prevalence of brushing ≥2 times/day. Across PPP quintiles, the prevalence of brushing <1 time/day decreased from 12.5% in Q1 to 4.4% in Q5, while brushing ≥2 times/day increased from 61.6 to 82.7% (Table 2; Figure 2A). Further multivariable regression models showed that sex, age group, WHO region, and PPP quintile were all associated with toothbrushing frequency (Supplementary Table S2).

**Table 2:** Prevalence of tooth brushing among adolescents aged 12–17 years, 2010–2019.

Group	Number of countries	<1 time				1 time				≥2 times			*p*-value
		Total	Boys	Girls	*p*-value	Total	Boys	Girls	*p*-value	Total	Boys	Girls	
Total	72	10.9 (10.0–11.8)	13.1 (12.0–14.3)	8.6 (7.7–9.6)	<0.001	18.7 (17.8–19.6)	21.2 (20.1–22.3)	16.1 (15.1–17.1)	<0.001	70.5 (68.8–72.0)	65.7 (63.9–67.4)	75.3 (73.6–77.0)	<0.001
Age													
12–14 years	72	12.2 (11.0–13.4)	14.3 (12.9–15.8)	10.1 (8.8–11.5)	<0.001	18.0 (16.8–19.3)	20.6 (19.1–22.1)	15.5 (14.2–16.8)	<0.001	69.8 (67.7–71.9)	65.1 (62.7–67.5)	74.5 (72.1–76.7)	<0.001
15–17 years	72	9.4 (8.4–10.4)	11.8 (10.5–13.2)	6.7 (5.9–7.7)	<0.001	19.5 (18.4–20.5)	21.9 (20.7–23.2)	16.8 (15.7–18.1)	<0.001	71.2 (69.5–72.8)	66.3 (64.4–68.1)	76.4 (74.6–78.2)	<0.001
*p*-value		<0.001	0.002	<0.001		0.042	0.125	0.082		0.229	0.363	0.128	
WHO region													
Africa	11	13.2 (11.9–14.6)	14.4 (12.9–16.1)	12.0 (10.7–13.4)	<0.001	24.2 (22.6–25.8)	27.4 (25.3–29.7)	20.9 (19.4–22.4)	<0.001	62.6 (60.8–64.4)	58.2 (55.9–60.4)	67.2 (65.2–69.1)	<0.001
Americas	19	5.3 (4.8–5.8)	6.3 (5.5–7.1)	4.4 (3.9–5.0)	<0.001	11.3 (10.5–12.2)	13.5 (12.5–14.6)	9.2 (8.4–10.1)	<0.001	83.3 (82.2–84.4)	80.2 (78.7–81.6)	86.4 (85.3–87.5)	<0.001
Eastern Mediterranean	14	30.7 (28.6–32.9)	35.3 (32.6–38.1)	25.7 (22.9–28.6)	<0.001	27.3 (25.5–29.0)	27.5 (25.2–30.0)	27.0 (24.9–29.1)	0.682	42.1 (40.0–44.2)	37.1 (34.6–39.7)	47.4 (44.5–50.3)	<0.001
South-East Asia	8	6.0 (5.4–6.8)	8.0 (7.0–9.1)	3.9 (3.4–4.6)	<0.001	16.9 (15.6–18.4)	20.3 (18.6–22.2)	13.3 (12.0–14.6)	<0.001	77.0 (75.2–78.8)	71.7 (69.2–74.0)	82.8 (81.1–84.4)	<0.001
Western Pacific	20	4.1 (3.2–5.2)	5.1 (4.2–6.1)	3.1 (2.1–4.5)	<0.001	14.6 (13.1–16.2)	16.9 (15.2–18.8)	12.4 (10.8–14.2)	<0.001	81.4 (79.2–83.4)	78.0 (75.7–80.1)	84.5 (81.9–86.8)	<0.001
*p*-value		<0.001	<0.001	<0.001		<0.001	<0.001	<0.001		<0.001	<0.001	<0.001	
PPP/capita, $													
Q1 100–3,299	14	12.5 (11.3–13.8)	13.8 (12.2–15.7)	10.8 (9.4–12.2)	0.003	25.8 (24.0–27.8)	28.2 (25.3–31.2)	22.8 (21.1–24.6)	0.001	61.6 (59.4–63.9)	58.0 (54.4–61.4)	66.4 (64.0–68.8)	<0.001
Q2 3,300–5,999	14	11.0 (9.7–12.4)	13.7 (12.0–15.7)	8.4 (7.3–9.6)	<0.001	24.7 (23.1–26.4)	27.0 (25.1–29.0)	22.5 (20.7–24.4)	<0.001	64.3 (62.0–66.6)	59.3 (56.5–62.0)	69.1 (66.6–71.5)	<0.001
Q3 6,000–9,999	15	17.0 (14.6–19.6)	19.3 (16.3–22.6)	14.7 (12.1–17.8)	0.006	16.5 (14.7–18.4)	17.5 (15.3–19.8)	15.5 (13.3–17.9)	0.100	66.5 (62.8–70.1)	63.3 (58.9–67.4)	69.8 (65.3–73.9)	0.002
Q4 10,000–17,999	15	6.0 (5.2–6.8)	8.3 (7.3–9.4)	3.7 (3.1–4.5)	<0.001	13.0 (11.9–14.1)	16.4 (15.1–17.8)	9.7 (8.6–10.8)	<0.001	81.1 (79.4–82.7)	75.3 (73.2–77.3)	86.6 (84.9–88.1)	<0.001
Q5 ≥ 18,000	14	4.4 (3.9–4.9)	6.2 (5.4–7.1)	2.6 (2.2–3.0)	<0.001	12.9 (12.2–13.6)	15.8 (14.9–16.7)	10.1 (9.4–10.9)	<0.001	82.7 (81.7–83.7)	78.0 (76.5–79.5)	87.3 (86.3–88.2)	<0.001
*p*-value		<0.001	<0.001	<0.001		<0.001	<0.001	<0.001		<0.001	<0.001	<0.001	

Data are presented as % (95%CI). WHO, World Health Organization; PPP/capita, purchasing power parity/capita.

### Hand hygiene

Table 3 presents the prevalence of inadequate hand hygiene (never or rarely washing before eating, after toilet use, and using soap) globally and by stratification variables. Overall, 6.5% (6.1–7.0) never or rarely washed hands before eating, 5.5% (5.1–5.9) after toilet use, and 9.0% (8.5–9.5) never or rarely used soap. Figures 1B–D illustrates the between-country variation in each hand hygiene indicator. Country-specific estimates are detailed in Supplementary Table S3, with the prevalence of inadequate handwashing before eating ranging from 1.7% (Laos) to 38.9% (Tuvalu), after toilet use from 1.6% (Lebanon) to 22.9% (Mauritania), and soap use from 2.2% (Lebanon) to 59.5% (Honduras). Boys showed higher prevalence of all three inadequate hand hygiene behaviours than girls. The largest sex difference was observed for soap use (10.4% vs. 7.6%). Older adolescents had a significantly higher prevalence of inadequate soap use than younger adolescents (10.1% vs. 8.0%), with no significant age difference for the other two behaviours (Table 3). Regional variation was also evident. The Region of the Americas and the Eastern Mediterranean Region had the highest prevalence of inadequate handwashing before eating (9.0 and 8.7%, respectively), whereas South-East Asia had the lowest (4.7%). The African and Eastern Mediterranean regions showed the highest prevalence of inadequate handwashing after toilet use (10.4 and 7.9%), and the Region of the Americas and African Region for inadequate soap use (14.3 and 13.9%). South-East Asia consistently showed the lowest prevalence of inadequate hand hygiene across all three indicators. The relationship between PPP and hand hygiene was less consistent than for toothbrushing (Table 3; Figures 2B–D). No clear stepwise pattern across quintiles was observed for handwashing before eating or after toilet use. For soap use, a U-shaped pattern was observed, with higher prevalence in both low-income (Q1: 10.0%) and high-income (Q5: 11.8%) countries relative to middle-income groups. Further multivariable regression models showed that sex, age group, PPP quintile, and WHO region were associated with inadequate hand hygiene outcomes (Supplementary Table S4).

**Table 3:** Prevalence of rarely or never wash hands among adolescents aged 12–17 years, 2010–2019.

Group	Number of countries	Before eating				After using the toilet				Using soap			
		Total	Boys	Girls	*p*-value	Total	Boys	Girls	*p*-value	Total	Boys	Girls	*p*-value
Total	72	6.5 (6.1–7.0)	6.9 (6.3–7.4)	6.2 (5.7–6.8)	0.052	5.5 (5.1–5.9)	6.5 (6.0–7.0)	4.5 (4.0–5.0)	0.003	9.0 (8.5–9.5)	10.4 (9.6–11.1)	7.6 (7.0–8.1)	0.003
Age													
12–14 years	72	6.3 (5.7–6.8)	6.7 (6.0–7.5)	5.8 (5.2–6.4)	0.017	5.5 (4.9–6.0)	6.4 (5.7–7.2)	4.5 (3.9–5.1)	<0.001	8.0 (7.4–8.6)	9.3 (8.6–10.2)	6.6 (6.1–7.3)	<0.001
15–17 years	72	6.9 (6.2–7.6)	7.0 (6.3–7.7)	6.7 (5.8–7.8)	0.583	5.5 (4.9–6.2)	6.5 (5.9–7.1)	4.5 (3.7–5.4)	<0.001	10.1 (9.3–11.0)	11.5 (10.5–12.6)	8.7 (7.7–9.7)	<0.001
*p-*value		0.179	0.625	0.079		0.863	0.887	0.989		<0.001	<0.001	<0.001	
WHO region													
Africa	11	6.6 (5.9–7.4)	7.1 (6.3–8.0)	6.1 (5.2–7.1)	0.040	10.4 (9.1–11.7)	11.1 (9.7–12.6)	9.6 (8.2–11.3)	0.071	13.9 (12.6–15.3)	15.1 (13.6–16.8)	12.7 (11.3–14.3)	0.004
Americas	19	9.0 (8.2–9.9)	8.9 (8.0–9.9)	9.1 (8.2–10.2)	0.658	5.8 (5.3–6.5)	6.2 (5.5–7.0)	5.4 (4.9–6.1)	0.019	14.3 (13.2–15.5)	14.7 (13.3–16.1)	13.9 (12.7–15.3)	0.305
Eastern Mediterranean	14	8.7 (7.8–9.6)	9.2 (8.0–10.7)	8.0 (7.0–9.2)	0.165	7.9 (7.0–8.9)	9.8 (8.5–11.3)	5.9 (4.8–7.1)	<0.001	9.4 (8.5–10.4)	12.2 (10.7–13.9)	6.3 (5.5–7.2)	<0.001
South-East Asia	8	4.7 (4.2–5.3)	4.9 (4.2–5.8)	4.5 (3.9–5.2)	0.383	3.4 (3.0–3.9)	4.3 (3.6–5.2)	2.5 (2.1–2.9)	<0.001	6.0 (5.2–6.8)	7.4 (6.1–8.8)	4.5 (3.9–5.0)	<0.001
Western Pacific	20	7.0 (5.7–8.6)	7.4 (6.1–8.9)	6.7 (5.1–8.7)	0.280	4.4 (3.3–5.9)	5.1 (4.1–6.4)	3.8 (2.6–5.6)	0.011	9.4 (8.1–10.7)	10.1 (9.0–11.4)	8.6 (7.2–10.4)	0.034
*p*-value		<0.001	<0.001	<0.001		<0.001	<0.001	<0.001		<0.001	<0.001	<0.001	
PPP/capita, $													
Q1 100–3,299	14	4.4 (3.7–5.3)	4.3 (3.2–5.7)	4.7 (4.1–5.4)	0.567	6.9 (5.9–7.9)	6.7 (5.4–8.4)	7.0 (6.0–8.2)	0.768	10.0 (8.6–11.6)	10.6 (8.4–13.4)	9.1 (8.0–10.4)	0.268
Q2 3,300–5,999	14	7.5 (6.7–8.5)	8.3 (7.2–9.6)	6.8 (5.9–7.9)	0.020	5.2 (4.6–5.9)	6.5 (5.8–7.4)	4.0 (3.4–4.7)	<0.001	12.0 (11.1–13.0)	13.3 (12.2–14.5)	10.7 (9.6–12.0)	<0.001
Q3 6,000–9,999	15	8.4 (7.1–10.0)	8.7 (7.4–10.3)	8.1 (6.4–10.3)	0.518	7.1 (5.8–8.6)	8.2 (6.9–9.8)	5.9 (4.5–7.8)	0.005	8.9 (7.7–10.3)	10.6 (9.3–12.2)	7.2 (5.8–9.0)	<0.001
Q4 10,000–17,999	15	5.6 (5.1–6.1)	6.1 (5.5–6.8)	5.1 (4.5–5.8)	0.024	3.7 (3.2–4.2)	4.9 (4.3–5.6)	2.5 (2.1–3.0)	<0.001	6.0 (5.5–6.5)	7.4 (6.7–8.2)	4.6 (4.1–5.1)	<0.001
Q5 ≥ 18,000	14	6.8 (6.3–7.3)	7.6 (7.0–8.2)	6.0 (5.5–6.6)	<0.001	5.3 (4.9–5.7)	6.2 (5.6–6.8)	4.4 (4.0–4.9)	<0.001	11.8 (11.1–12.5)	13.9 (13.0–14.8)	9.7 (8.9–10.5)	<0.001
*p*-value		<0.001	<0.001	<0.001		<0.001	<0.001	<0.001		<0.001	<0.001	<0.001	

Data are presented as % (95%CI).

## Discussion

This study presents a comprehensive, up-to-date assessment of oral and hand hygiene practices among adolescents aged 12–17 years across 72 countries. We found that although the majority of adolescents brushed their teeth at least once daily, nearly 1 in 10 did so less than once per day, and almost 30% fell short of the recommended twice-daily frequency. Inadequate hand hygiene was also common, with never or rarely using soap (9.0%) being the most prevalent deficiency, followed by never or rarely washing hands before eating (6.5%) and after toilet use (5.5%). Large disparities were observed across all examined stratification variables, with boys, younger adolescents, and those residing in the Eastern Mediterranean and African regions bearing a disproportionate burden. Notably, toothbrushing showed a strong monotonic economic gradient, with prevalence of inadequate brushing decreasing steadily as country income rose, whereas hand hygiene practices did not follow a simple income gradient.

Our estimate of brushing <1 time/day (10.9%) is slightly lower than the 13.0% reported by Das Gupta et al. (9) for never/rarely brushing, which may reflect differences in country coverage or age range. Both studies identified the Eastern Mediterranean Region as having the highest prevalence of inadequate brushing and the Region of the Americas as having the best performance, lending external validity to our regional estimates. For hand hygiene, our findings are broadly consistent with Jatrana et al. (10), who reported that only 30.3% of adolescents practised appropriate hand hygiene (washing with soap, after toilet use, and before eating). Accordingly, their estimate of adolescents not always washing with soap (40.7%) is, as expected, higher than our “never or rarely” estimate (9.0%), reflecting the different cutoffs. Our estimates are also consistent with previous GSHS-based studies in the Caribbean (15). These cross-study consistencies underscore the value of standardised global surveillance systems in tracking adolescent hygiene trends and guiding resource allocation across diverse settings.

The consistent finding that boys have poorer hygiene than girls across all four indicators has been reported in other GSHS-based studies (9, 16). Das Gupta et al. found that in 69 of 72 countries (95.8%), boys were more likely to never or rarely brush their teeth (9). Similarly, a global study on hand hygiene observed the same pattern (10, 17). Several mechanisms might contribute to this disparity. Because the GSHS does not collect data on gender socialisation, health-seeking behaviour, or risk-taking tendencies, these explanations are drawn from existing literature and remain speculative. It has been suggested that girls may receive more intensive socialisation regarding cleanliness and appearance, may exhibit higher health-seeking behaviours, and may be less prone to risk-taking behaviours that lead to neglect of preventive practices (18). These observations raise the possibility that hygiene promotion programmes could usefully target boys through gender-sensitive messaging, male-role models, and boy-friendly educational materials to narrow this persistent gap (19, 20).

Younger adolescents (12–14 years) had poorer oral hygiene but slightly better hand hygiene (for soap use) than older adolescents. The poorer oral hygiene among younger adolescents might reflect less established routines and lower awareness of the long-term consequences of poor oral health (21, 22). Conversely, the higher prevalence of inadequate soap use among older adolescents (10.1% vs. 8.0%) could potentially be explained by increased autonomy and reduced parental supervision, as well as by a potential decline in compliance with health recommendations during adolescence – a pattern also observed for physical activity and seatbelt use (23). These patterns suggest that oral hygiene interventions might usefully prioritise early adolescence to establish durable habits, whereas hand hygiene programmes for older adolescents might benefit from incorporating peer-led strategies and school-based reminder systems to sustain compliance as parental oversight diminishes.

The extremely high prevalence of inadequate brushing in the Eastern Mediterranean Region (30.7%) warrants particular attention. This region includes countries with protracted conflicts (Syria, Yemen, and Palestine), economic hardship, and cultural practices that may not recognise the miswak (traditional chewing stick) as “brushing,” potentially leading to underreporting (24, 25). The GSHS does not, however, capture data on traditional oral hygiene practices, so this explanation is inferred from regional qualitative studies and cannot be directly verified in our dataset. Even considering miswak use, the oral hygiene situation in this region remains concerning. The high prevalence of never/rarely using soap in the Americas (14.3%) appeared to be largely accounted for by Honduras (59.5%), an extreme outlier. The reasons for this exceptionally low soap use require further investigation – possible factors include limited access to soap, inadequate handwashing facilities in schools, or inadequate health education (26, 27). Because the GSHS does not collect information on product availability, sanitation infrastructure, or school health education, these explanations are hypothetical and require dedicated investigation. These regional and country-specific patterns suggest that contextually adapted interventions may be warranted: in the Eastern Mediterranean Region, oral health programmes could usefully respect local cultural practices while promoting effective hygiene behaviours, and in settings such as Honduras, policy action may be needed to ensure universal access to soap and functional handwashing facilities in schools.

The strong monotonic gradient for toothbrushing – improving steadily with country income – is consistent with the expectation that toothbrushes and toothpaste are consumer goods that become more affordable as income rises (28, 29). However, the lack of a clear gradient for hand hygiene was unexpected, given that soap is generally inexpensive, yet we found that the prevalence of never/rarely using soap was actually higher in the richest quintile (11.8%) than in the poorest (10.0%). One possible explanation is that in high-income countries, alcohol-based hand sanitisers may replace soap, possibly leading to underreporting of soap use without implying inadequate hygiene (30, 31). We acknowledge that the GSHS questionnaire does not distinguish between soap and other hand-cleansing products, so this interpretation remains speculative. Alternatively, the question specifically asks about “using soap when washing your hands”–in some high-income settings, liquid soap or handwash products may not be perceived as “soap” by respondents. Cultural and social desirability biases may also contribute (32). This finding highlights the need for caution when interpreting self-reported soap use across diverse cultural contexts, and suggests that hand hygiene improvement strategies may need to be tailored to local behavioural and economic contexts rather than relying solely on income-driven development.

### Implications for policy and practice

Because this study is cross-sectional, the following implications are derived from observed prevalence patterns rather than evidence of intervention effectiveness. The findings suggest several directions for consideration. School-based hygiene promotion programmes could be warranted in low-income countries and in the Eastern Mediterranean and African regions, where the burden of inadequate hygiene is highest (33, 34). Interventions might usefully consider engaging boys, who lag behind girls across all indicators—potentially through boy-friendly educational materials, male peer educators, and integration of hygiene messages into sports-related activities (35, 36). For oral hygiene, promoting twice-daily toothbrushing with fluoridated toothpaste may be a relevant target (37). For hand hygiene, promoting soap use (or alcohol-based sanitisers where soap is unavailable) and handwashing before eating and after toilet use could be considered for concurrent promotion, as deficiencies for each behaviour vary by region (38). Given the weak economic gradient for hand hygiene, behaviour-change interventions (e.g., hygiene education, reminder systems) may warrant consideration alongside economic development (32). The extremely low soap use in some countries (e.g., Honduras) warrants country-specific investigations to identify local barriers such as lack of soap availability, inadequate handwashing facilities, or low perceived effectiveness (26).

### Strengths and limitations

This study has several strengths. It uses the largest and most recent standardised dataset on adolescent hygiene behaviours, covering 72 countries and 244,040 individuals. The GSHS sampling and weighting procedures ensure national representativeness, and the recalculation of sampling weights ensures equal country contribution to pooled estimates, preventing over-representation of populous nations and yielding more accurate prevalence profiles within each stratum. The simultaneous examination of four hygiene indicators allows direct comparison of different behaviours within the same population. Several limitations should be acknowledged. First, all data are self-reported and subject to recall and social desirability bias, although the anonymous questionnaire likely reduces overreporting of desirable behaviours (39, 40). Second, the GSHS only includes school-attending adolescents, who may have better hygiene practices than out-of-school youth; our findings thus likely represent an upper bound of hygiene performance (41). Third, we could not adjust for individual-level socioeconomic status because household wealth data were unavailable (42). Fourth, the hand hygiene questions refer to “washing hands” without specifying whether water availability; in water-scarce settings, respondents may never have the opportunity to wash, but the questionnaire does not capture this constraint (4). Fifth, the GSHS employs a cross-sectional design, which precludes any inference of causality or temporal sequence between the examined correlates and hygiene practices. Sixth, the GSHS does not collect information on existing national preventive programmes (e.g., school-based caries prevention, hygiene education campaigns) or on the availability of oral hygiene products and handwashing infrastructure (water, soap, and sanitary facilities) in participating countries at the time of survey administration. These contextual factors likely influenced the observed between-country differences but could not be incorporated into our analytical models. Seventh, country-level clustering was not modelled through random-effects multilevel analysis; the Complex Samples adjustment applies to school-level nesting within countries only. Eighth, surveys spanned 2010–2019. We assumed hygiene behaviours remained stable over this period; although survey year was adjusted for in the multivariable models, secular trends could not be fully excluded, and between-country comparisons may partly reflect temporal variation. Ninth, approximately 12–13% of respondents had missing data on at least one hygiene indicator and were excluded via listwise deletion. While this may introduce selection bias if missingness was related to hygiene practices, the magnitude of missingness is modest and unlikely to substantially alter the observed prevalence patterns.

## Conclusion

Inadequate oral and hand hygiene practices are common among adolescents globally, with notable disparities by sex, age, region, and country income level. While the majority brush their teeth at least once daily, nearly 30% do not achieve twice-daily brushing, and 1 in 10 rarely uses soap when washing hands. Boys, younger adolescents, and those in low-income and Eastern Mediterranean countries are priority populations for interventions. School-based hygiene promotion programmes that are gender-sensitive, age-appropriate, and adapted to local contexts hold promise for improving hygiene behaviours and reducing the burden of oral and infectious diseases.

## Data Availability

The datasets presented in this study can be found in online repositories. The names of the repository/repositories and accession number(s) can be found at: https://extranet.who.int/ncdsmicrodata/index.php/catalog/GSHS/?page=1&from=2021&to=2021&ps=15&repo=GSHS.
