# Supplementary material for: Oral and hand hygiene practices among adolescents aged 12–17 years in 72 countries: a cross-sectional study using global school-based student health survey data (2010–2019)
Source: Front Public Health. 2026 Sep 10;14:1939346. doi: 10.3389/fpubh.2026.1939346 (PMC13601238; doi:10.3389/fpubh.2026.1939346)
Supplement: Supplementary file 1 [file Table_1.docx]

Table S1. Prevalence of tooth brushing among adolescents aged 12-17 years by countries, 2010-2019

| Country | <1 time |  | 1 time |  | ≥2 times |
| --- | --- | --- | --- | --- | --- |
| **Africa** |  |  |  |  |  |
| Algeria | 20.8(18.1-23.8) |  | 20.3(18.8-21.9) |  | 58.9(55.3-62.4) |
| Benin | 2.6(1.9-3.6) |  | 24.8(21.0-29.2) |  | 72.5(68.4-76.3) |
| Eswatini | 4.6(3.7-5.7) |  | 30.1(27.7-32.7) |  | 65.3(62.7-67.8) |
| Ghana | 9.4(7.6-11.6) |  | 28.8(25.1-32.8) |  | 61.8(58.5-65.0) |
| Liberia | 12.3(9.5-15.6) |  | 17.3(14.8-20.2) |  | 70.4(67.0-73.6) |
| Mauritania | 15.2(13.2-17.5) |  | 21.7(18.5-25.1) |  | 63.1(58.1-68.0) |
| Mauritius | 4.9(3.8-6.2) |  | 30.0(27.7-32.5) |  | 65.1(62.6-67.5) |
| Mozambique | 5.9(4.2-8.1) |  | 13.5(11.2-16.3) |  | 80.6(77.0-83.8) |
| Namibia | 11.2(9.2-13.6) |  | 24.0(20.9-27.4) |  | 64.8(60.6-68.9) |
| Sierra Leone | 7.7(5.9-9.9) |  | 21.5(18.1-25.5) |  | 70.8(66.7-74.5) |
| Tanzania | 14.2(11.8-17.1) |  | 26.6(23.6-29.9) |  | 59.2(55.5-62.7) |
| **Americas** |  |  |  |  |  |
| Bahamas | 6.0(4.7-7.5) |  | 20.7(17.9-23.7) |  | 73.4(70.6-76.0) |
| Barbados | 3.5(2.7-4.6) |  | 9.1(7.5-11.1) |  | 87.4(85.2-89.3) |
| Belize | 2.2(1.6-2.9) |  | 7.7(6.3-9.3) |  | 90.1(88.3-91.7) |
| Bolivia | 10.8(9.7-12.1) |  | 19.7(18.2-21.2) |  | 69.5(67.3-71.7) |
| Curacao | 3.7(3.0-4.5) |  | 21.7(19.1-24.5) |  | 74.7(72.1-77.1) |
| Dominican Republic | 5.1(3.2-8.1) |  | 9.1(7.1-11.6) |  | 85.7(82.3-88.6) |
| El Salvador | 2.8(1.9-4.0) |  | 7.9(6.4-9.7) |  | 89.3(87.0-91.2) |
| Guatemala | 5.8(4.2-7.8) |  | 5.4(3.7-7.7) |  | 88.9(85.8-91.3) |
| Guyana | 4.4(3.4-5.7) |  | 13.2(11.5-15.0) |  | 82.5(79.9-84.7) |
| Honduras | 4.1(3.2-5.3) |  | 8.0(6.8-9.5) |  | 87.8(86.1-89.4) |
| Panama | 2.5(1.8-3.4) |  | 10.5(9.0-12.3) |  | 87.0(85.1-88.7) |
| Paraguay | 2.5(2.0-3.1) |  | 6.8(5.7-8.1) |  | 90.7(89.2-92.0) |
| Peru | 4.4(3.7-5.4) |  | 13.0(11.4-14.8) |  | 82.5(80.5-84.4) |
| Saint Kitts and Nevis | 3.8(2.9-4.7) |  | 86.7(85.1-88.3) |  | 9.5(8.1-10.9) |
| Saint Lucia | 4.9(3.7-6.5) |  | 12.6(10.9-14.5) |  | 82.5(80.4-84.5) |
| Saint Vincent and the Grenadines | 3.4(2.7-4.4) |  | 14.7(12.8-16.9) |  | 81.8(79.6-83.9) |
| Suriname | 3.2(2.2-4.7) |  | 10.9(9.4-12.7) |  | 85.9(83.4-88.0) |
| Trinidad and Tobago | 4.9(3.3-7.2) |  | 17.1(15.3-19.2) |  | 77.9(75.4-80.3) |
| Uruguay | 2.5(2.0-3.1) |  | 9.1(8.2-10.2) |  | 88.3(87.1-89.5) |
| **Eastern Mediterranean** |  |  |  |  |  |
| Afghanistan | 26.7(20.2-34.4) |  | 33.5(30.0-37.2) |  | 39.8(32.7-47.4) |
| Bahrain | 14.0(11.4-17.1) |  | 23.2(21.9-24.6) |  | 62.8(59.7-65.7) |
| Egypt | 38.4(32.8-44.4) |  | 29.1(24.4-34.4) |  | 32.4(27.5-37.8) |
| Iraq | 22.4(19.6-25.5) |  | 27.8(25.8-29.8) |  | 49.8(46.3-53.3) |
| Kuwait | 15.0(11.3-19.6) |  | 25.9(23.5-28.4) |  | 59.1(53.2-64.7) |
| Lebanon | 8.3(7.2-9.4) |  | 26.3(24.0-28.6) |  | 65.5(62.8-68.0) |
| Morocco | 33.0(29.7-36.4) |  | 21.6(19.8-23.4) |  | 45.4(42.4-48.5) |
| Oman | 13.1(11.9-14.3) |  | 79.7(78.3-81.1) |  | 7.3(6.4-8.2) |
| Palestine | 28.4(26.4-30.6) |  | 22.9(22.0-23.8) |  | 48.7(46.2-51.2) |
| Qatar | 19.0(15.2-23.5) |  | 16.8(14.6-19.3) |  | 64.2(59.0-69.0) |
| Sudan | 10.0(7.7-12.8) |  | 37.3(32.5-42.4) |  | 52.7(47.2-58.2) |
| Syria | 33.5(30.5-36.6) |  | 26.1(24.1-28.2) |  | 40.4(37.1-43.8) |
| United Arab Emirates | 12.1(9.5-15.4) |  | 24.6(22.4-26.9) |  | 63.3(59.2-67.2) |
| Yemen | 35.2(31.1-39.5) |  | 26.7(23.7-30.0) |  | 38.1(34.3-42.0) |
| **South-East Asia** |  |  |  |  |  |
| Bangladesh | 12.8(10.8-15.2) |  | 23.6(20.2-27.4) |  | 63.5(58.6-68.2) |
| Bhutan | 7.5(6.8-8.4) |  | 50.3(48.1-52.6) |  | 42.1(39.7-44.6) |
| Indonesia | 2.2(1.9-2.7) |  | 8.5(7.4-9.8) |  | 89.2(87.8-90.5) |
| Myanmar | 6.0(4.9-7.4) |  | 33.4(30.1-36.9) |  | 60.6(57.0-64.0) |
| Nepal | 14.1(11.2-17.7) |  | 37.1(33.6-40.8) |  | 48.7(43.9-53.6) |
| Sri Lanka | 2.9(2.4-3.6) |  | 27.6(24.1-31.4) |  | 69.5(65.6-73.1) |
| Thailand | 5.1(4.0-6.4) |  | 11.0(9.5-12.8) |  | 83.9(81.5-86.0) |
| Timor-Leste | 14.6(12.5-17.0) |  | 13.6(11.9-15.5) |  | 71.8(68.7-74.8) |
| **Western Pacific** |  |  |  |  |  |
| Brunei Darussalam | 3.2(2.3-4.3) |  | 8.7(7.2-10.5) |  | 88.1(86.3-89.8) |
| Cambodia | 4.4(3.2-6.0) |  | 16.6(13.7-19.9) |  | 79.0(75.0-82.6) |
| Cook Islands | 12.2(9.8-15.0) |  | 23.6(20.2-27.4) |  | 64.2(59.6-68.6) |
| Fiji | 3.0(2.2-4.1) |  | 12.2(10.4-14.2) |  | 84.8(82.6-86.8) |
| French Polynesia | 7.3(6.0-8.9) |  | 15.0(13.5-16.6) |  | 77.7(75.8-79.5) |
| Kiribati | 18.7(15.2-22.8) |  | 16.3(14.1-18.7) |  | 65.0(60.8-69.1) |
| Laos | 2.3(1.5-3.5) |  | 9.3(7.3-11.7) |  | 88.4(85.4-90.9) |
| Malaysia | 2.7(2.3-3.1) |  | 10.6(9.9-11.4) |  | 86.7(85.8-87.6) |
| Mongolia | 5.1(4.3-6.0) |  | 27.8(26.2-29.4) |  | 67.1(65.2-69.0) |
| Nauru | 11.4(8.7-14.1) |  | 60.9(56.7-65.1) |  | 27.7(23.8-31.6) |
| Niue | 9.8(4.7-14.9) |  | 80.5(73.8-87.2) |  | 9.8(4.7-14.9) |
| Philippines | 5.6(3.7-8.5) |  | 9.4(7.6-11.7) |  | 85.0(80.2-88.7) |
| Samoa | 3.8(2.6-5.4) |  | 11.9(9.8-14.4) |  | 84.3(81.5-86.8) |
| Solomon Islands | 23.0(19.3-27.1) |  | 18.4(15.4-21.7) |  | 58.6(54.0-63.1) |
| Tokelau | 17.0(12.0-23.5) |  | 17.5(7.3-36.1) |  | 65.6(50.2-78.3) |
| Tonga | 7.3(6.1-8.6) |  | 12.9(11.6-14.3) |  | 79.8(77.9-81.6) |
| Tuvalu | 7.1(5.4-8.8) |  | 60.6(57.4-63.8) |  | 32.3(29.2-35.4) |
| Vanuatu | 10.1(8.6-11.7) |  | 16.4(14.0-19.2) |  | 73.5(70.6-76.2) |
| Vietnam | 2.9(2.4-3.6) |  | 20.7(18.1-23.5) |  | 76.4(73.4-79.2) |
| Wallis and Futuna | 8.5(6.7-10.8) |  | 11.6(9.8-13.7) |  | 79.9(77.1-82.4) |

Table S2. Associations of sex, age group, WHO region, and country-level purchasing power parity (PPP) quintile with toothbrushing frequency among adolescents aged 12–17 years

|  | <1 time | |  | 1 time | |  | ≥2 times | |
| --- | --- | --- | --- | --- | --- | --- | --- | --- |
|  | *OR(95%CI)* | *P value* |  | *OR(95%CI)* | *P value* |  | *OR(95%CI)* | *P value* |
| Sex |  |  |  |  |  |  |  |  |
| Girls | Ref. |  |  | Ref. |  |  | Ref. |  |
| Boys | 1.63(1.48-1.80) | <0.001 |  | 1.36(1.27-1.46) | <0.001 |  | 0.62(0.58-0.67) | <0.001 |
| Age |  |  |  |  |  |  |  |  |
| 12-14 years | Ref. |  |  | Ref. |  |  | Ref. |  |
| 15-17 years | 0.93(0.91-0.96) | <0.001 |  | 1.13(1.05-1.22) | <0.001 |  | 0.95(0.88-1.03) | 0.198 |
| PPP category, $ |  |  |  |  |  |  |  |  |
| Q1 100–3299 | Ref. |  |  | Ref. |  |  | Ref. |  |
| Q2 3300–5999 | 0.58(0.49-0.69) | <0.001 |  | 0.99(0.87-1.14) | 0.222 |  | 1.20(1.05-1.38) | <0.001 |
| Q3 6000–9999 | 0.96(0.78-1.17) | 0.349 |  | 0.62(0.52-0.73) | <0.001 |  | 1.32(1.11-1.58) | <0.001 |
| Q4 10000–17,999 | 0.42(0.35-0.51) | <0.001 |  | 0.43(0.37-0.51) | <0.001 |  | 2.71(2.32-3.18) | <0.001 |
| Q5 ≥ 18,000 | 0.37(0.31-0.45) | <0.001 |  | 0.56(0.48-0.65) | <0.001 |  | 2.28(1.96-2.65) | <0.001 |
| **WHO region** |  |  |  |  |  |  |  |  |
| Africa | 3.21(2.46-4.19) | <0.001 |  | 1.70(1.45-1.98) |  |  | 0.41(0.35-0.49) | <0.001 |
| Americas | 1.18(0.89-1.59) | 0.150 |  | 0.95(0.82-1.11) | 0.132 |  | 1.03(0.84-1.25) | 0.640 |
| Eastern Mediterranean | 9.22(7.10-11.97) | <0.001 |  | 2.40(2.10-2.74) | <0.001 |  | 0.16(0.14-0.19) | <0.001 |
| South-East Asia | 1.88(1.42-2.49) | <0.001 |  | 1.57(1.34-1.84) | <0.001 |  | 0.53(0.44-0.62) | <0.001 |
| Western Pacific | Ref. |  |  | Ref. |  |  | Ref. |  |

Adjusted for sex, age, PPP, region, and survey year.

Table S3. Prevalence of rarely or never wash hands among adolescents aged 12-17 years by countries, 2010-2019

| Country | Before eating |  | After using the toilet |  | Using soap |
| --- | --- | --- | --- | --- | --- |
| **Africa** |  |  |  |  |  |
| Algeria | 5.7(4.9-6.8) |  | 2.9(2.3-3.7) |  | 5.4(4.6-6.4) |
| Benin | 4.5(3.4-6.1) |  | 12.4(8.3-18.3) |  | 22.5(17.9-27.8) |
| Eswatini | 4.5(3.3-6.2) |  | 3.8(3.3-4.4) |  | 13.8(12.3-15.4) |
| Ghana | 7.0(4.6-10.5) |  | 7.7(5.9-10.0) |  | 10.6(9.0-12.4) |
| Liberia | 10.0(8.0-12.5) |  | 6.2(4.9-7.9) |  | 7.9(6.1-10.3) |
| Mauritania | 7.1(5.7-8.8) |  | 22.9(17.0-30.1) |  | 12.5(9.5-16.4) |
| Mauritius | 34.2(32.0-36.4) |  | 10.5(8.9-12.4) |  | 23.2(21.3-25.2) |
| Mozambique | 7.4(5.5-10.0) |  | 6.2(4.0-9.4) |  | 9.8(6.5-14.6) |
| Namibia | 4.8(3.9-5.9) |  | 6.2(5.1-7.4) |  | 10.6(8.8-12.7) |
| Sierra Leone | 6.5(5.2-8.1) |  | 6.6(5.0-8.6) |  | 11.5(9.1-14.3) |
| Tanzania | 7.0(5.9-8.3) |  | 17.4(14.8-20.4) |  | 21.1(18.9-23.5) |
| **Americas** |  |  |  |  |  |
| Bahamas | 15.9(13.9-18.1) |  | 5.1(3.8-6.8) |  | 9.0(7.5-10.8) |
| Barbados | 14.2(11.9-16.8) |  | 1.7(1.1-2.4) |  | 7.9(6.5-9.5) |
| Belize | 3.6(2.8-4.4) |  | 1.6(1.2-2.1) |  | 4.7(3.7-6.0) |
| Bolivia | 15.9(14.3-17.7) |  | 10.8(9.2-12.5) |  | 22.7(20.5-25.1) |
| Curacao | 12.3(10.7-14.1) |  | 3.7(2.9-4.6) |  | 6.2(5.2-7.5) |
| Dominican Republic | 14.1(10.2-19.1) |  | 4.7(3.2-6.9) |  | 8.8(6.9-11.2) |
| El Salvador | 5.2(4.1-6.6) |  | 4.0(2.8-5.5) |  | 5.8(4.7-7.1) |
| Guatemala | 4.6(3.3-6.4) |  | 2.7(2.0-3.6) |  | 5.8(4.6-7.3) |
| Guyana | 9.8(8.1-11.9) |  | 6.3(4.8-8.2) |  | 11.7(9.7-14.0) |
| Honduras | 7.4(6.4-8.5) |  | 5.2(4.0-6.6) |  | 59.5(56.3-62.7) |
| Panama | 14.5(12.2-17.2) |  | 5.2(3.9-6.8) |  | 9.2(7.8-10.9) |
| Paraguay | 7.9(6.7-9.1) |  | 2.6(2.0-3.3) |  | 6.1(5.1-7.3) |
| Peru | 5.3(4.2-6.6) |  | 6.7(5.6-8.0) |  | 8.1(6.6-9.8) |
| Saint Kitts and Nevis | 11.8(10.3-13.3) |  | 2.9(2.1-3.7) |  | 6.6(5.4-7.8) |
| Saint Lucia | 19.0(16.8-21.4) |  | 5.6(4.4-7.0) |  | 11.4(9.5-13.5) |
| Saint Vincent and the Grenadines | 19.4(17.5-21.4) |  | 4.6(3.6-5.9) |  | 12.8(11.3-14.5) |
| Suriname | 11.4(9.4-13.8) |  | 5.1(3.8-6.8) |  | 9.2(7.8-10.7) |
| Trinidad and Tobago | 16.0(13.8-18.5) |  | 4.2(2.6-6.6) |  | 12.5(10.2-15.4) |
| Uruguay | 12.8(11.2-14.6) |  | 7.0(5.9-8.4) |  | 3.7(2.9-4.8) |
| **Eastern Mediterranean** |  |  |  |  |  |
| Afghanistan | 5.6(3.7-8.3) |  | 5.3(3.5-7.9) |  | 11.6(7.5-17.4) |
| Bahrain | 8.3(7.6-9.1) |  | 4.1(3.4-5.0) |  | 4.1(3.5-4.9) |
| Egypt | 12.8(10.5-15.5) |  | 9.4(7.0-12.4) |  | 7.8(5.7-10.6) |
| Iraq | 6.8(5.5-8.5) |  | 8.9(7.0-11.4) |  | 4.1(3.1-5.4) |
| Kuwait | 10.9(9.2-12.8) |  | 7.1(6.0-8.5) |  | 7.9(6.0-10.3) |
| Lebanon | 4.8(4.1-5.7) |  | 1.6(1.2-2.0) |  | 2.2(1.8-2.6) |
| Morocco | 4.9(4.3-5.6) |  | 6.1(5.3-7.1) |  | 10.7(9.4-12.2) |
| Oman | 7.7(6.8-8.6) |  | 8.6(7.6-9.6) |  | 7.0(6.1-7.9) |
| Palestine | 7.2(6.3-8.2) |  | 5.6(4.8-6.6) |  | 7.1(6.1-8.2) |
| Qatar | 17.4(13.4-22.3) |  | 15.3(11.8-19.7) |  | 17.1(13.1-21.9) |
| Sudan | 7.8(6.6-9.2) |  | 12.0(10.4-13.8) |  | 22.6(18.8-26.9) |
| Syria | 9.4(7.7-11.4) |  | 3.7(2.5-5.6) |  | 6.6(5.0-8.7) |
| United Arab Emirates | 8.4(7.4-9.7) |  | 3.1(2.4-4.1) |  | 3.6(2.9-4.4) |
| Yemen | 7.0(5.4-8.9) |  | 10.5(9.1-12.1) |  | 15.4(13.4-17.6) |
| **South-East Asia** |  |  |  |  |  |
| Bangladesh | 2.9(1.7-4.9) |  | 2.1(1.0-4.3) |  | 5.5(3.2-9.5) |
| Bhutan | 3.7(3.1-4.5) |  | 4.3(3.8-4.9) |  | 5.9(5.1-6.7) |
| Indonesia | 2.5(2.0-3.2) |  | 2.4(1.9-2.9) |  | 3.9(3.2-4.6) |
| Myanmar | 6.7(5.5-8.1) |  | 8.4(6.8-10.2) |  | 5.7(4.8-6.9) |
| Nepal | 3.8(2.8-5.2) |  | 4.3(3.3-5.7) |  | 4.7(3.5-6.3) |
| Sri Lanka | 2.4(1.8-3.3) |  | 2.7(2.0-3.5) |  | 6.5(5.4-7.9) |
| Thailand | 15.9(13.3-18.8) |  | 6.4(5.0-8.1) |  | 15.0(13.4-16.6) |
| Timor-Leste | 16.9(15.0-19.1) |  | 22.4(19.4-25.7) |  | 15.9(13.8-18.4) |
| **Western Pacific** |  |  |  |  |  |
| Brunei Darussalam | 2.7(2.2-3.4) |  | 2.6(2.0-3.3) |  | 8.2(7.1-9.5) |
| Cambodia | 2.0(1.4-2.8) |  | 2.9(2.1-3.8) |  | 2.5(1.9-3.3) |
| Cook Islands | 7.2(5.2-9.8) |  | 4.4(2.9-6.7) |  | 6.0(4.2-8.5) |
| Fiji | 3.9(3.1-4.9) |  | 2.4(1.8-3.2) |  | 5.9(4.9-7.0) |
| French Polynesia | 8.7(7.4-10.3) |  | 5.3(4.1-6.8) |  | 11.4(10.0-13.1) |
| Kiribati | 21.4(17.4-26.0) |  | 16.3(13.5-19.4) |  | 16.5(14.3-18.9) |
| Laos | 1.7(1.1-2.5) |  | 3.4(2.5-4.7) |  | 7.7(6.4-9.2) |
| Malaysia | 5.0(4.5-5.5) |  | 5.3(4.8-5.9) |  | 13.6(12.8-14.5) |
| Mongolia | 7.4(6.5-8.4) |  | 11.0(9.6-12.5) |  | 3.1(2.5-3.7) |
| Nauru | 7.3(5.1-9.5) |  | 6.0(4.0-8.0) |  | 8.4(6.0-10.8) |
| Niue | 6.8(2.5-11.1) |  | 3.0(0.1-5.9) |  | 3.7(0.5-6.9) |
| Philippines | 7.7(5.0-11.8) |  | 6.4(4.0-10.1) |  | 8.0(5.6-11.3) |
| Samoa | 18.4(15.7-21.5) |  | 6.8(5.3-8.7) |  | 14.0(11.7-16.7) |
| Solomon Islands | 7.2(4.7-10.8) |  | 8.6(6.6-11.1) |  | 10.3(7.1-14.8) |
| Tokelau | 14.7(10.8-19.6) |  | 4.9(1.9-12.2) |  | 8.2(5.1-12.9) |
| Tonga | 14.7(13.0-16.5) |  | 7.4(6.3-8.6) |  | 19.7(18.0-21.6) |
| Tuvalu | 38.9(35.7-42.1) |  | 17.9(15.4-20.4) |  | 20.8(18.1-23.5) |
| Vanuatu | 4.7(3.5-6.1) |  | 5.3(4.1-6.7) |  | 7.1(5.7-8.7) |
| Vietnam | 7.7(6.2-9.5) |  | 2.1(1.5-2.8) |  | 10.3(8.8-12.1) |
| Wallis and Futuna | 12.9(10.7-15.4) |  | 7.5(5.8-9.5) |  | 8.5(6.6-10.8) |

Table S4. Associations of sex, age group, WHO region, and country-level purchasing power parity (PPP) quintile with inadequate hand hygiene (never or rarely washing hands before eating, after toilet use, or using soap) among adolescents aged 12–17 years

|  | Before eating | |  | After using the toilet | |  | Using soap | |
| --- | --- | --- | --- | --- | --- | --- | --- | --- |
|  | *OR(95%CI)* | *P value* |  | *OR(95%CI)* | *P value* |  | *OR(95%CI)* | *P value* |
| Sex |  |  |  |  |  |  |  |  |
| Girls | Ref. |  |  | Ref. |  |  | Ref. |  |
| Boys | 1.63(1.48-1.80) | <0.001 |  | 1.36(1.27-1.46) | <0.001 |  | 0.62(0.58-0.67) | <0.001 |
| Age |  |  |  |  |  |  |  |  |
| 12-14 years | Ref. |  |  | Ref. |  |  | Ref. |  |
| 15-17 years | 0.93(0.91-0.96) | <0.001 |  | 1.13(1.05-1.22) | <0.001 |  | 0.95(0.88-1.03) | 0.198 |
| PPP category, $ |  |  |  |  |  |  |  |  |
| Q1 100–3299 | Ref. |  |  | Ref. |  |  | Ref. |  |
| Q2 3300–5999 | 0.58(0.49-0.69) | <0.001 |  | 0.99(0.87-1.14) | 0.222 |  | 1.20(1.05-1.38) | <0.001 |
| Q3 6000–9999 | 0.96(0.78-1.17) | 0.349 |  | 0.62(0.52-0.73) | <0.001 |  | 1.32(1.11-1.58) | <0.001 |
| Q4 10000–17,999 | 0.42(0.35-0.51) | <0.001 |  | 0.43(0.37-0.51) | <0.001 |  | 2.71(2.32-3.18) | <0.001 |
| Q5 ≥ 18,000 | 0.37(0.31-0.45) | <0.001 |  | 0.56(0.48-0.65) | <0.001 |  | 2.28(1.96-2.65) | <0.001 |
| **WHO region** |  |  |  |  |  |  |  |  |
| Africa | 3.21(2.46-4.19) | <0.001 |  | 1.70(1.45-1.98) |  |  | 0.41(0.35-0.49) | <0.001 |
| Americas | 1.18(0.89-1.59) | 0.150 |  | 0.95(0.82-1.11) | 0.132 |  | 1.03(0.84-1.25) | 0.640 |
| Eastern Mediterranean | 9.22(7.10-11.97) | <0.001 |  | 2.40(2.10-2.74) | <0.001 |  | 0.16(0.14-0.19) | <0.001 |
| South-East Asia | 1.88(1.42-2.49) | <0.001 |  | 1.57(1.34-1.84) | <0.001 |  | 0.53(0.44-0.62) | <0.001 |
| Western Pacific | Ref. |  |  | Ref. |  |  | Ref. |  |

Adjusted for sex, age, PPP, region, and survey year.
